# Effect of Nano-SiO_2_ on the Hydration, Microstructure, and Mechanical Performances of Solid Waste-Based Cementitious Materials

**DOI:** 10.3390/ma18112636

**Published:** 2025-06-04

**Authors:** Zian Geng, Yu Zhang, Yiwen Zhou, Jiapeng Duan, Zhuqing Yu

**Affiliations:** 1 College of Materials Science and Engineering, Nanjing Tech University, Nanjing 211816, China; 2 State Key Laboratory of Materials-Oriented Chemical Engineering, Nanjing 211816, China

**Keywords:** solid waste-based cementitious materials, nano-SiO_2_, mechanical properties, chloride diffusion coefficient, microstructure

## Abstract

Solid waste-based cementitious materials (SWBC) are composed of steel slag (SS), granulated blast furnace slag (GBFS), fly ash (FA), desulfurization gypsum (DG), and Portland cement (PC). Currently, SWBC holds great potential as a sustainable building material; however, its low early compressive strength and volume expansion limit its range of application. Therefore, the main objective of this study is to enhance the mechanical properties and dimensional stability of SWBC by adding nano-SiO_2_, while also improving its resistance to chloride ions, thereby promoting its use in the field of sustainable building materials. A comprehensive experimental approach integrating mechanical performance testing, shrinkage analysis, and chloride diffusion coefficient evaluation was established, with the testing methods of thermogravimetric analysis-differential scanning calorimetry (TG-DSC), X-ray diffraction (XRD), and scanning electron microscopy (SEM). The study found that adding nano-SiO_2_ enhanced the nucleation of calcium silicate hydrates (C-S-H) gel in hydrated SWBC, leading to improved compressive strength and reduced chloride permeability when SiO_2_ addition was 0.5%. When the hydration period extends to 28 days, the modified SWBC achieves a compressive strength of 56 MPa. However, excessive nano-SiO_2_ (≥1%) inhibited the long-term hydration of SWBC but had no significant effect on the final compressive strength.

## 1. Introduction

Portland cement (PC) is extensively employed across a multitude of construction endeavors, driven by the rapid pace of economic development and societal progress. However, the production and utilization of PC will cause certain environmental problems [1,2]. For instance, the PC production process consumes a large amount of resources and generates a significant number of waste gases, including carbon dioxide and carbon monoxide [1,3,4]. Research findings [5,6] demonstrate that each ton of PC manufacturing releases 650 kg of CO_2_ alongside suspended particulates and gaseous pollutants (e.g., CO, NO_x_, SO_x_), constituting significant environmental externalities. With the application of environmental protection policies, an increasing number of scholars are seeking alternatives to PC to achieve resource conservation and environmental protection.

Concurrently, the development of industrial processes has accumulated a large amount of solid waste, becoming an urgent global environmental challenge. Solid waste is typically divided into three main categories: alkaline waste, including materials like red mud, calcium carbide slag, and alkali slag; sulfate waste, encompassing desulfurization gypsum (DG) and electrolytic manganese slag; and silicon-aluminum waste, which comprises granular blast furnace slag (GBFS), fly ash (FA), and steel slag (SS) [7,8]. Industrial solid wastes are characterized by their substantial generation volumes, wide-ranging types, diverse physicochemical properties, and heterogeneous spatial distribution [9,10,11].

Solid waste-based cementitious materials (SWBC) are cementitious substances primarily derived from industrial solid waste [12]. Through controlled proportioning and hydration-activated mechanisms, these industrial byproducts can develop cementitious matrices, exhibiting extensive applicability in structural engineering, geotechnical stabilization, and mineral resource recovery systems [13,14,15,16,17]. The main characteristics of hydration in SWBC are sulfate activation and depolymerization-repolymerization process [18,19]. Sulfate activation refers to the process where sulfate ions in the SWBC act as activators to accelerate the hydration reaction of cementitious components [18]. The depolymerization-repolymerization process involves the breaking down of existing silicate chains and their subsequent reformation into more stable and denser structures, which enhances the mechanical attributes and durability of the SWBC [19]. Contrasted with PC, the production process of SWBC exhibits lower carbon emissions. Official data indicate that the industry that produces PC accounts for approximately 7% to 8% of CO_2_ emissions. Conversely, SWBC mainly uses industrial byproducts like FA and GBFS, reducing dependence on natural resources and significantly lowering CO_2_ emissions [20].

However, SWBC exhibits inherent limitations. SWBC faces critical technical challenges due to its multi-component composition (GBFS, FA, etc.). The chemical composition of these solid wastes is different from that of PC. The proportion of ettringite (AFt) in the hydration products is relatively high, reducing the quality of the building. Due to the inhomogeneity of solid waste components, different hydration rates and internal stress development occur, which compromise the volume stability of SWBC and increase the risk of cracking [21]. These variations can result in microcracks and subsequent structural degradation, especially under severe environmental conditions. To address these issues, nano-SiO_2_ has a certain modifying effect on SWBC.

Nano-SiO_2_ possesses significant reactivity and surface activity, effectively enhancing the cement hydration process [22]. Utilizing nano-SiO_2_ to modify cementing materials can enhance the matrix by optimizing the particle size and dosage of SiO_2_, which facilitates the reaction between GGBS and Ca(OH)_2_ [23,24]. Meanwhile, the high specific surface area of nano-SiO_2_ offers nucleation locations for the generation of hydration products, enhancing the development of calcium silicate hydrates (C-S-H) gel [22,25]. However, nano-SiO_2_ has a relatively high specific surface area and small particle size. When it is incorporated into SWBC at a high level, it is prone to problems such as agglomeration. Therefore, the incorporation amount with nano-SiO_2_ needs to be limited to a certain extent, and the incorporation amount is usually below 3% [25,26,27].

Despite significant advancements in the field of SWBC, several knowledge gaps still exist. The current research mainly focuses on the individual influence of different components on cement-based materials, while there is a lack of research on SWBC with a relatively high solid waste content. Furthermore, although some studies have explored the influence of nano-SiO_2_ on the performance of SWBC, there are relatively few studies on the long-term mechanical properties and durability of nano-SiO_2_ modified SWBC. This study aims to address these technical challenges associated with low early mechanical and poor volume stability in SWBC, which arise from its multi-component composition, by investigating the mechanical and micro-structural properties of nano-SiO_2_ modified SWBC through a comprehensive experimental approach that integrates mechanical performance testing, shrinkage analysis, and chloride diffusion coefficient evaluation. By filling these knowledge gaps, this research not only offers valuable insights and practical direction for the development of eco-friendly and high-performance cementitious materials but also provides a scientific basis for their wider application in various engineering contexts.

## 2. Materials and Methods

### 2.1. Materials

**Figure 1:**
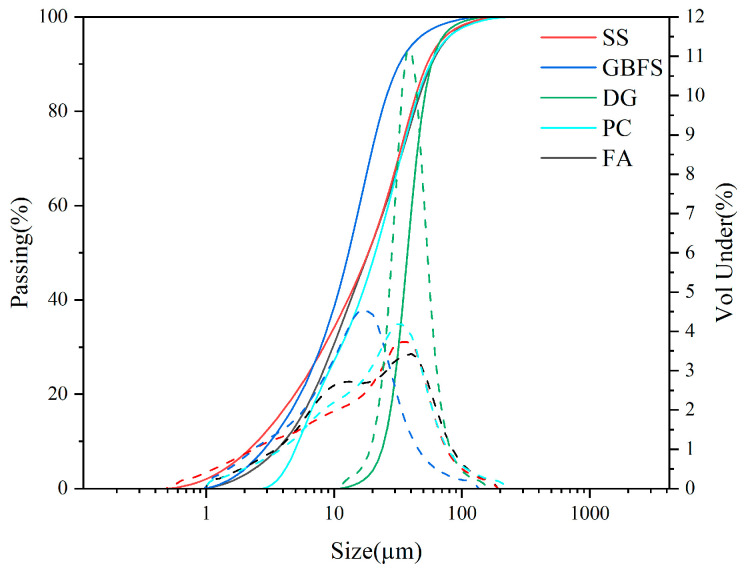
The particle size distribution of SS, GBFS, DG, PC, and FA.

**Figure 2:**
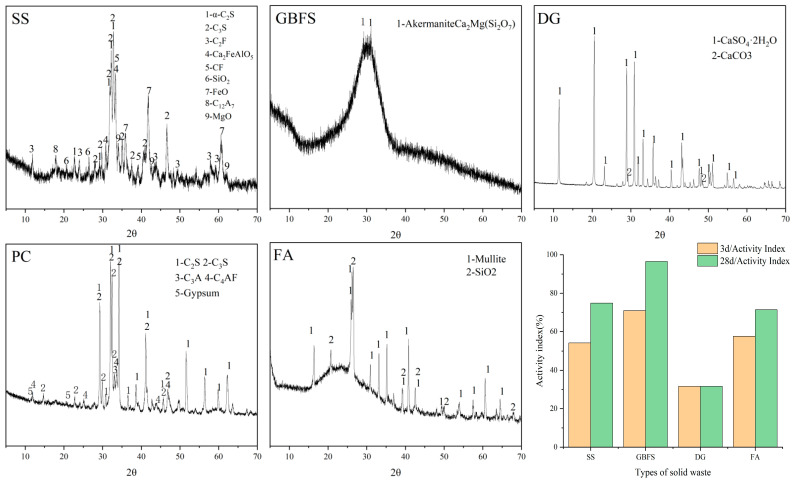
XRD patterns and activity index of SS, GBFS, DG, PC, and FA.

Raw materials, including SS, GBFS, DG, PC, and FA, are commercially available products in the market. Table 1 displays the chemical compositions of the raw materials. The particle size analysis of the solid waste materials used is shown in Figure 1. The major mineral compositions and material activity of SS, GBFS, DG, PC, and FA are shown in Figure 2, the main mineral composition of GBFS was the amorphous phase, since GBFS accounted for the highest proportion of the components, this resulted in the preparation of SWBC with an amorphous phase as the main mineral component. All raw materials were screened through a 75-micron sieve. Nano-SiO_2_ in Table 2 characterized by particle dimensions ranging from 7 to 40 nm and a definite surface area of 150 m^2^/g, was procured from Shanghai Aladdin Biochemical Technology Co., Ltd. (Shanghai, China).

Because nano-SiO_2_ tends to agglomerate, it was first mixed with mixing water, then ultrasonically dispersed in an ultrasonic cleaning instrument for 10 min, and subsequently mixed with the solid waste material.

The composition design of the paste ratio is shown in Table 3. Adjust the dosage of GBFS, DG, and PC, and design the composition ratio of A, B, and C. Nano-SiO_2_ was added to the paste in the form of external addition, with mass contents of 0%, 0.5%, 1.0%, and 2.0%, maintaining a persistent water-to-cement ratio of 0.3.

### 2.2. Preparation of the Samples

Binder pastes were prepared with a water-to-binder ratio of 0.3. The raw materials were initially dry-mixed using a mechanical mixer for one minute to ensure uniform blending. Then, the dispersed nano-SiO_2_ solution was added to the mixture and blended for an additional four minutes to ensure uniformity. Put the mixture into a mold in size of 20 mm × 20 mm × 20 mm. The molds were subjected to vibration using a vibration table and subsequently cured in a standard curing box at 20 ± 2 °C and 95 ± 2% relative humidity for 24 h. Following demolding, the specimens were cured in water at 20 °C for testing at intervals of 3, 7, 28, 90, and 180 days.

### 2.3. Test Methods

The specimens’ compressive strength was assessed following GB/T 17671-2021 [28], with curing periods of 3, 7, 28, 90, and 180 days under standard conditions. The measurement of compressive strength was conducted utilizing a universal testing machine, with the load applied at a rate of 2.4 KN per second. The results were obtained from the average of the three samples at each curing age.

The length change was measured following the Chinese standard GB/T 29417-2012 [29]. Before the test, specimens were taken out of the water and their surfaces were wiped to dry. The length of the specimens was tested using a comparator with an accuracy of 0.001. The length change (expansion) ratio is shown in (1):

(1) $$E_{x}=\frac{L_{x}-L_{1}}{L_{0}}\times 100$$

where *E_x_* is the linear expansion ratio (%), *L_x_* is the length at X days (mm), *L*_1_ is the length after molding (mm), *L*_0_ is the effective length of the specimen and the value is 80 mm in this test. Three specimens were measured and the average expansion ratio was calculated.

X-ray diffraction(XRD) analysis was utilized to ascertain the mineral phases present in the samples. The XRD patterns were obtained through an automated diffractometer equipped with a Cu tube, operated at 40 kilovolts (kV) and 30 milliamperes (mA). Data collection spanned a 2θ angle range from 5 to 65 degrees, with readings taken at each 0.01-degree increment for one second per step. These measurements were carried out at ambient temperature with the samples held in a stationary position.

In the process of quantitative X-ray diffraction (QXRD) analysis, samples were mixed with titanium dioxide (TiO_2_) at 20 wt.% as an internal standard to assist in quantifying crystalline and amorphous contents [30,31]. The hydration product analysis was performed using the XRD-Rietveld method, which involved refining various parameters such as zero-shift errors, background polynomial parameters, phase scale factors, unit cell parameters, peak shape parameters, and preferred orientation coefficients [32,33,34]. During the quantitative analysis, the weighted R-profile was kept below 10 to ensure accuracy.

The thermal analyzer operates within a temperature range of 25 °C to 1000 °C, employing a ceramic crucible to perform simultaneous differential thermal and thermogravimetric analyses under a nitrogen atmosphere, with a consistent heating rate maintained at 10 °C/min. The mass of C-S-H (*M_C-S-H_*) can be determined using the following formula:

(2) $$M_{C\text{-}S\text{-}H}=\frac{{\mathrm{BW}}_{\mathrm{Total}}-\left({\mathrm{BW}}_{\mathrm{AFt}}\times \omega_{\mathrm{AFt}}\right)}{\omega_{C\text{-}S\text{-}H}}$$

where *BW_Total_* is the total weight loss between 50 and 300 °C, *BW_AFt_* is the AFt mass quantified by XRD, *ω_AFt_* is the bound water percentage in AFt (approximately 45.9% [35,36]), *ω_C-S-H_* is the proportion of bound water within C-S-H gel (approximately 20% [35,36]).

Scanning electron microscopy (SEM) was employed to examine the morphological features of the fractured, unpolished samples. Prior to SEM imaging, the samples underwent gold coating via a sputtering process to augment their electrical conductivity.

The chloride ion diffusion coefficient was measured following the GB/T 42272-2022 [37] standard. Specimens with dimensions of Φ100 mm × 50 mm were crafted and tested using the Rapid Chloride Migration (RCM) method. The measurement age was selected as 56 days to account for the high GBFS content in SWBC.

(3) $$D_{\mathrm{RCM}}=\frac{0.0239\left(273+T\right)L}{\left(U-2\right)t}\left(X_{d}-0.0238\sqrt{\frac{\left(273+T\right)LX_{d}}{U-2}}\right)$$

where *D_RCM_* is the concrete chloride migration coefficient (m^2^/s), *U* is the absolute value of the applied voltage (V), *T* is the average of the initial and ending temperatures of the anode solution (°C), *L* is the thickness of the specimen (mm), *X_d_* is the mean chloride ion penetration depth, measured in millimeters and precise to 0.1 mm, *t* is the duration of test runs (h).

## 3. Results and Discussion

### 3.1. Compressive Strength

**Figure 3:**
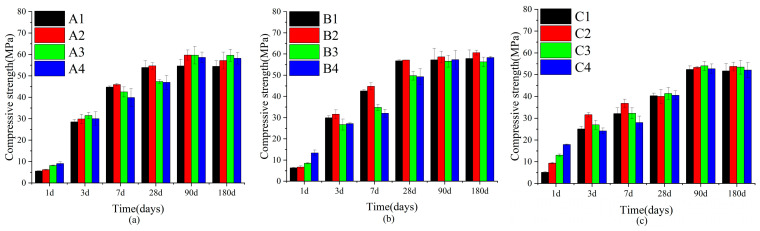
Variations in the compressive strength of SWBC due to varying nano-SiO_2_ contents over time: (**a**) Set A; (**b**) Set B; (**c**) Set C.

In Figure 3, the compressive strength of SWBC exhibited a continuously increasing trend with the increase of the curing age. From the early stages at 1 day and 3 days, through to 7 days, and then extending to the later stages at 28 days, 90 days, and 180 days, the compressive strength of each formulation had mostly experienced a gradual increase. This was primarily attributed to the fact that as time elapsed, the hydration reactions within the material persisted, and the hydration products increased. Meanwhile, the adjustment of PC and GBFS also affected the later strength to a certain extent. This was also why, compared with groups A and B, the intensity of group C was significantly lower than that of groups A and B.

From the overall trend, the addition of nano-SiO_2_ significantly enhanced the compressive strength of SWBC materials. By comparing the control groups (A1, B1, C1) in each formula with the groups that had nano-SiO_2_ added (A2, B2, C2, etc.), it was found that the addition of nano-SiO_2_ had significantly improved the strength of the material at all ages. Groups A2, B2, and C2, which incorporated a nano-SiO_2_ content of 0.5%, all exhibited the highest compressive strength at most of the ages observed. This was due to the fact that nano-SiO_2_, with its high specific surface area and high activity, could effectively promote hydration reactions and fill the internal pores of the material, thereby enhancing the density and strength of the material [38,39,40].

Compared with the research of Cai [41], the optimal dosage of nano-SiO_2_ in this study was smaller. This was mainly due to the proportioning design and the treatment method. In the three groups, the content of PC was lower and the content of GBFS was higher, resulting in less Ca(OH)_2_ generated internally and limiting the reaction of nano-SiO_2_. Meanwhile, in some experiments, the addition of water-reducing agents can regulate the fluidity of the slurry, enabling it to achieve better working performance at a higher dosage.

When the incorporation amount of nano-SiO_2_ was too high, in the early hydration stage, nano-SiO_2_ greatly promoted the development of hydration. However, as the hydration time extended, the number of nanoparticles in the mixture exceeded the amount needed to bind with free particles in the hydration products. This led to the agglomeration of nano-SiO_2_, the formation of microcracks, and the leaching of a portion of nano-SiO_2_, ultimately resulting in insufficient strength of the SWBC [42,43]. At the same time, nano-SiO_2_ had extremely high hydrophilicity, absorbing some of the free water, which led to insufficient water in the hydration reaction and delayed the hydration development of SWBC. This resulted in lower strength for groups A3, A4, B3, B4, C3, and C4 at 3–28 days, but the strength became comparable after 90 days.

### 3.2. Autogenous Shrinkage Setup

**Figure 4:**
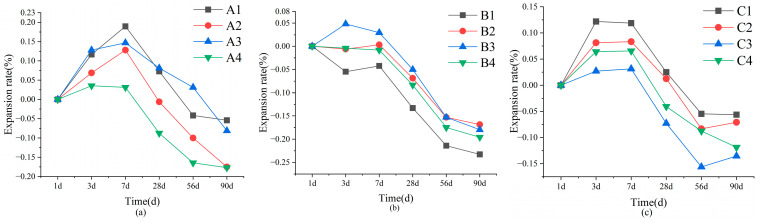
Variations in the linear change in SWBC due to varying nano-SiO_2_ contents over time: (**a**) Set A; (**b**) Set B; (**c**) Set C.

In Figure 4, SWBC exhibits more pronounced linear expansion changes. By adjusting the mix ratio of SWBC, their linear expansion could reach 0.2%. Therefore, it was essential to consider the linear change in SWBC. Unlike PC, the volume change in SWBC was affected by multiple factors [2,44]. The hydration product of DG, AFt, induced volume expansion in SWBC, whereas a high dosage of GBFS caused pore formation, leading to volumetric contraction [45,46]. As shown in Figure 4, the incorporation of DG significantly affected the linear expansion of SWBC, which was one of the reasons for the higher linear expansion in Group A. In the comparison of the design of Groups A and C, it was evident that Group C exhibited a smaller linear change. The formation of AFt necessitates the consumption of the aluminate phase [47]. Given that Group C had a lower FA content than Group A, the efficiency of AFt formation was reduced in Group C. Concurrently, GBFS induced volumetric contraction in the cementitious material [45,46].

Nano-SiO_2_ demonstrated a notable modifying effect on cementitious materials. In Figure 4, nano-SiO_2_ in Groups A, B, and C effectively reduced the linear change rate of the cementitious material, thereby reducing its linear shrinkage. The incorporation of 0.5% and 2% nano-SiO_2_ significantly influenced SWBC. The addition of 0.5% nano-SiO_2_ enhances the formation of C-S-H gel and postpones the development and conversion of AFt, thereby minimizing its effect on SWBC [48]. Nano-SiO_2_ has high hydrophilicity. When the content of nano-SiO_2_ was too high, the internal moisture was adsorbed by nano-SiO_2_, which slowed down the hydration process of SWBC [41]. However, it did not affect its ultimate strength. This is also why 2% nano-SiO_2_ could effectively reduce the linear change in SWBC.

### 3.3. X-Ray Diffraction Analysis

**Figure 5:**
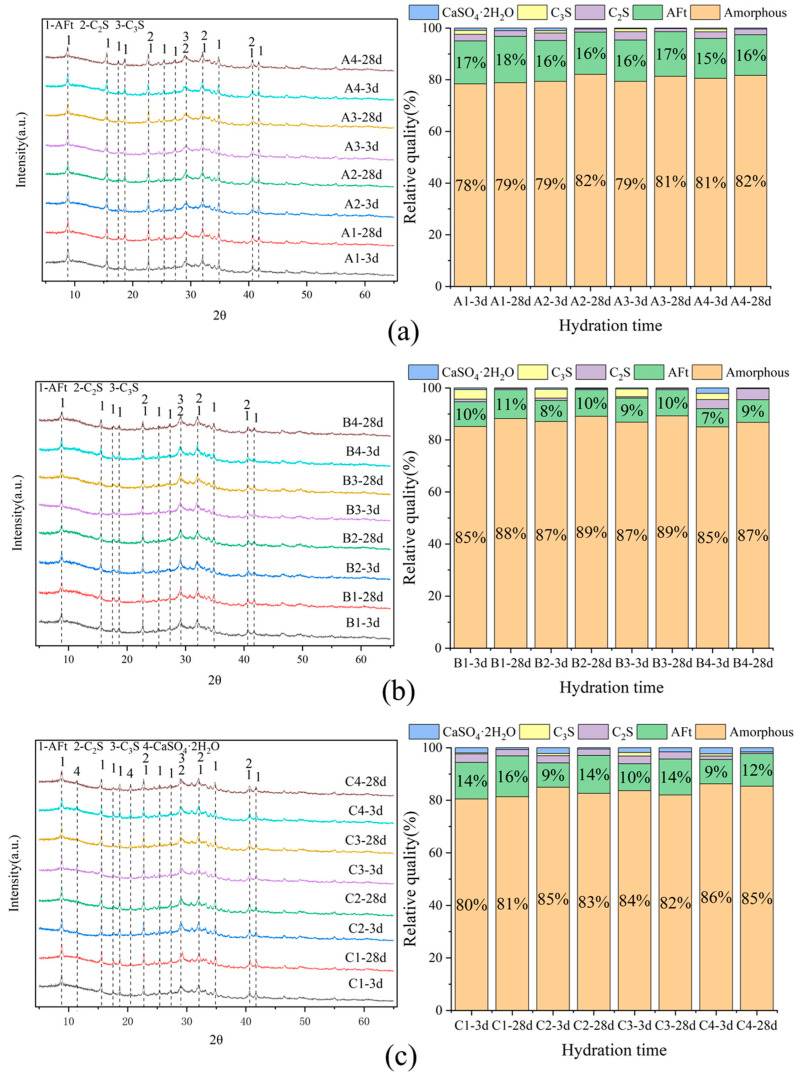
Composition analysis of hydrated 3 days and 28 days in SWBC with different proportions of nano-SiO_2_: (**a**) Set A; (**b**) Set B; (**c**) Set C.

Figure 5 shows that the primary peaks were AFt, C_2_S, and CaSO_4_·2H_2_O, with SWBC hydration products including AFt and C-S-H gel [25,49]. The low Ca(OH)_2_ content in SWBC hydration products is mainly due to the generation of Ca(OH)_2_ from the hydration of SS and PC under alkaline conditions, while FA particles provide reactive SiO_2_ and Al_2_O_3_. The reaction with Ca(OH)_2_ and DG in the system led to the formation of AFt, significantly decreasing the Ca(OH)_2_ content in the hydration products [50]. In SWBC, certain impurities did not participate in the hydration process. However, due to their relatively low incorporation amounts, coupled with the high content of amorphous materials in the cementitious materials, the peaks of these substances were not significant in the XRD patterns.

The XRD quantitative analysis mainly identified the formation of CaSO_4_·2H_2_O, C_2_S, AFt, and amorphous phases. It can be seen from the XRD quantitative chart that the amorphous gel accounted for the majority of the hydration products of SWBC. This is mainly because the amorphous content in GBFS powder was extremely high, accounting for more than 95%, and the amorphous content in SS and FA was relatively high, accounting for about 40% [36]. In Figure 5, Group A had a higher proportion of AFt, while Groups B and C had similar AFt content. This is mainly because the formation of AFt is influenced by DG and PC. The Ca^2+^ and SO_4_^2−^ ions produced by the dissolution of DG can react with C_3_A or Ca(OH)_2_ to form ettringite [51].

Figure 5 demonstrates that the addition of nano-SiO_2_ altered the hydration efficiency of silicon and aluminum phases, as well as the optimal sulfur content [47,52]. The incorporation of nano-SiO_2_ into SWBC did not alter the types of its hydration products. Nano-SiO_2_ negatively affected the hydration rate of DG. Negatively charged nano-SiO_2_ particles adhere to the positively charged aluminate phase via electrostatic bridging, which slows the consumption of DG and the formation of Aft [53]. Moreover, when the amount of DG was relatively low, the inhibitory effect of nano-SiO_2_ was more pronounced [22,54,55].

### 3.4. Thermogravimetric Analysis of Hydrated Samples

The composition of hydration products can be determined through thermogravimetric analysis. Using thermogravimetric analysis-differential scanning calorimetry (TG-DSC) to analyze the main dehydration temperature of hydration products, its main components can be analyzed. The primary hydration products of SWBC were C-S-H gel and Aft [25,49]. The water content of C-S-H gel is approximately 20% [36], whereas AFt has a significantly higher water content of 45.9% [36]. The dehydration temperature range for C-S-H gel spans from 50 °C to 600 °C, primarily involving the elimination of interlayer water and hydroxyl groups within its structure [36]. AFt is a hexagonal prism-shaped crystal, in which multiple aluminum oxygen octahedra [Al(OH)_6_]^3−^ are connected by calcium ions and hydroxyl ions to form a columnar structure [Ca_6_·Al_2_(OH)_12_]^6+^[3SO_4_^2−^·26H_2_O]^6−^ [36]. Sulfate ions and water molecules are positioned on the exterior of the aluminum oxygen column. Water molecules located between the aluminum oxygen columns in AFt are removed at approximately 100 °C, whereas the water produced from the dehydroxylation of aluminum hydroxide octahedra is lost between 200 °C and 400 °C [36]. The content of hydration products in SWBC was ultimately determined through the quantitative analysis of AFt by XRD and the proportion of water loss by TG.

**Figure 6:**
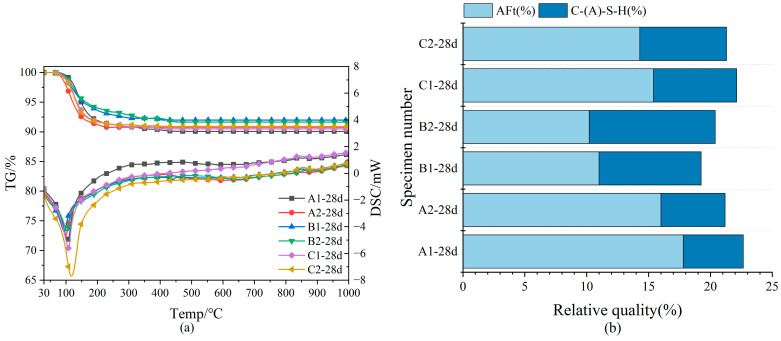
TG-DSC analysis of nano-SiO_2_ mixed with SWBC and relative mass percentage of hydration products: (**a**) TG-DSC graph; (**b**) Proportion of hydration products.

In Figure 6a, from the TG-DSC curves of the late hydrated samples, the main peak of DSC occurred around 100 °C. With the rising temperature, the DSC data exhibited no notable peaks, indicating that the predominant hydration products in SWBC were C-S-H and AFt and that the majority of internal DG had been hydrated and depleted by day 28 [25]. In the TG data, the primary mass loss of SWBC occurred below 400 °C, aligning with the main dehydration temperature range of C-S-H gel and AFt previously discussed. The water loss of SWBC was relatively small, mainly around 9%. The absence of a minor endothermic peak around 455 °C in Groups A, B, and C suggests a low proportion of Ca(OH)_2_ in the hydration products of SWBC.

Nano-SiO_2_ decreased AFt formation while promoting C-S-H gel development. Figure 6b demonstrates that nano-SiO_2_ significantly enhances the formation of C-S-H in Group B, with B2 exhibiting a notably higher quantity than B1. In Groups A and C, the formation of C-S-H remains largely unchanged, while AFt generation is inhibited. Nano-SiO_2_ enhances the activity of GBFS and, when added appropriately, reacts with Ca(OH)_2_ from SWBC hydration to form additional C-S-H gel, thereby promoting the hydration of solid waste materials [56].

### 3.5. Micro-Morphology of the Hardened Pastes

**Figure 7:**
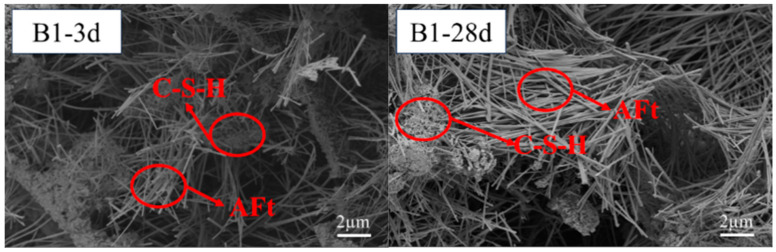
Notably, 3d and 28d SEM images of SWBC without nano-SiO_2_.

**Figure 8:**
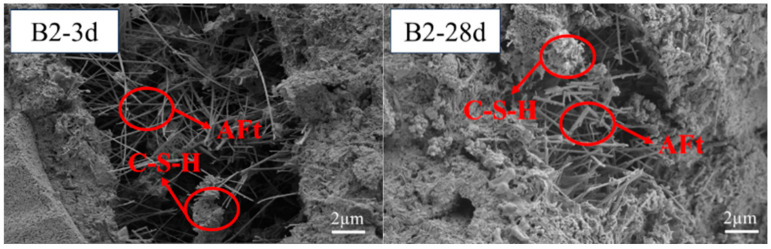
Notably, 3 days and 28 days SEM images of SWBC with 0.5% nano-SiO_2_.

Figure 7 and Figure 8 display micro-pores, primarily a result of the cementitious material’s heterogeneous hydration process. The hydration of SWBC started from some fragments. The hydration of sulfates was relatively fast, and there was “flash setting”. Some hydration products were generated within the first ten minutes of hydration [57]. With ongoing hydration, the hydration products gradually appeared at the top of the dehydrated particles and gradually enveloped the particles, forming aggregates [57]. In the later stage of hydration, thick layers were formed, resulting in a solid structure [57]. Figure 7 and Figure 8 display micro-pores, primarily a result of the cementitious material’s heterogeneous hydration process. The hydration of SWBC started from some fragments. The hydration of sulfates was relatively fast, and there was a “flash setting”. Some hydration products were generated within the first ten minutes of hydration [57]. With ongoing hydration, the hydration products gradually appeared at the top of the dehydrated particles and gradually enveloped the particles, forming aggregates [57]. In the later stage of hydration, thick layers were formed, resulting in a solid structure [57].

The hydration samples contained numerous needle-like structures, identified as AFt, a primary hydration product of SWBC. It was formed by the combination of DG and Ca(OH)_2_ from other hydration products. AFt formation significantly bolstered the early strength of the samples during hydration [58].

### 3.6. Chloride Diffusion Coefficient

Nano-silica has a certain improvement effect on the chloride ion diffusion coefficient of cement-based cementitious materials [51,59]. Testing the chloride ion diffusion coefficient of the modified SWBC can expand its application range and lay a foundation for later research.

**Figure 9:**
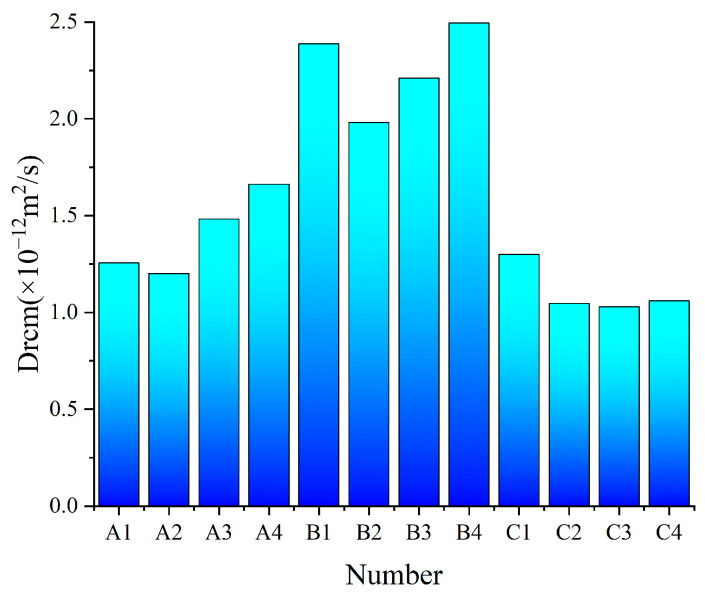
nano-SiO_2_ effect of chloride ion diffusion coefficient on SWBC.

Given the slow hydration rate of SWBC, the chloride ion diffusion coefficient was tested at 56 days. Figure 9 demonstrates that the chloride ion diffusion coefficient of SWBC is less than 3 × 10^−12^ m^2^/s. Analysis of chloride ion diffusion coefficients revealed that Group B exhibited a higher coefficient compared to other groups.

The ability of SWBC to hinder chloride ion diffusion could be attributed to the following aspects: In SWBC, the addition of GBFS and FA significantly reduces porosity and refines the cementitious matrix’s pore structure [60]. The formation of C-S-H gel adsorbs chloride ions, obstructing their diffusion path [61]. In SWBC with FA and GBFS, the concentration of ions like Ca^2+^, Al^3+^, AlOH^2+^, and Si^4+^ exceeds that in PC, yet their diffusion capacity is reduced, potentially restricting chloride ion migration [62]. FA and GBFS contain higher levels of C_3_A, enabling greater adsorption of chloride ions and the formation of Friedel’s salt [62].

The incorporation of nano-SiO_2_ inhibited the chloride diffusion coefficient in SWBC, with a pronounced effect in group B, but less so in groups A and C. This is attributed to nano-SiO_2_ enhancing C-S-H formation, and the higher PC content in group B supplying more Ca(OH)_2_ during hydration, leading to increased C-S-H gel content and improved resistance to chloride ion penetration [25,48,53]. When the nano-SiO_2_ content was too high, nano-SiO_2_ was easy to agglomerate, with hydration consumption, the remaining pores in SWBC. Conversely, the slowed hydration development led to reduced erosion resistance in SWBC [25,48,53].

## 4. Conclusions

The study underscores the promising application of nano-SiO_2_-modified SWBC in sustainable construction. According to the experimental results of XRD, TG, SEM analysis, and thermodynamic modeling, the main conclusions could be drawn as follows:

(1) The mechanical properties of SWBC are found to be optimal when the incorporation amount of nano-SiO_2_ is 0.5%. Although excessive incorporation of nano-SiO_2_ (≥1%) slows down the hydration rate of SWBC, it does not have a negative impact on the ultimate strength. Furthermore, nano-SiO_2_ significantly mitigates the linear variation of SWBC, with this effect being particularly pronounced at higher incorporation levels.
(2) The incorporation of nano-SiO_2_ promotes C-S-H gel formation and partially delays AFt development. The inhibitory effect on AFt formation becomes more pronounced with increasing nano-SiO_2_ content. The impact of nano-SiO_2_ on SWBC is modulated to some extent by its proportion in the mix.
(3) The incorporation of nano-SiO_2_ exerts a certain influence on the corrosion resistance of SWBC. Incorporating 0.5% nano-SiO_2_ significantly improves the corrosion resistance of SWBC and decreases its chloride diffusion coefficient. Excessive nano-SiO_2_ dosage can negatively affect the corrosion resistance of SWBC.

This research lays the foundation for experimental directions such as the later SWBC ratio regulation, durability evaluation under different environments, and environmental impact assessment, and broadens the thinking for the application of SWBC.

## Figures and Tables

**Table 1:** Chemical composition of experimental raw materials.

	SS	GBFS	DG	PC	FA
CaO	48.57	40.41	32.06	62.13	3.43
SiO_2_	17.89	29.87	1.65	21.75	51.13
Al_2_O_3_	1.86	16.70	0.79	5.21	29.67
MgO	5.36	8.71	0.70	2.09	0.78
Fe_2_O_3_	16.88	0.28	0.20	2.91	5.07
K_2_O	0.05	0.35	0.08	0.63	2.20
SO_3_	0.21	0	42.46	1.97	1.16
P_2_O_5_	0	0	0.01	0	0.33
LOI	21.69	0.65	21.59	1.80	4.08

**Table 2:** Properties of nano-SiO_2_.

Item	Diameter (nm)	Surface-Volume Ratio (m^2^/g)	Purity (%)
Target	7–40	150	99.8%

**Table 3:** Composition design of paste ratio (%).

	SS	GBFS	DG	PC	FA	Nano-SiO_2_
A1	10	65	10	10	5	0
A2	10	65	10	10	5	0.5
A3	10	65	10	10	5	1
A4	10	65	10	10	5	2
B1	10	65	5	15	5	0
B2	10	65	5	15	5	0.5
B3	10	65	5	15	5	1
B4	10	65	5	15	5	2
C1	10	70	10	5	5	0
C2	10	70	10	5	5	0.5
C3	10	70	10	5	5	1
C4	10	70	10	5	5	2

## Data Availability

The original contributions presented in this study are included in the article. Further inquiries can be directed to the corresponding authors.
